# A comparison of low-dose risperidone to paroxetine in the treatment of panic attacks: a randomized, single-blind study

**DOI:** 10.1186/1471-244X-9-25

**Published:** 2009-05-26

**Authors:** James M Prosser, Samantha Yard, Annie Steele, Lisa J Cohen, Igor I Galynker

**Affiliations:** 1 The Department of Psychiatry and Behavioral Sciences, Beth Israel Medical Center, Albert Einstein College of Medicine, First Ave at 16th St, New York, NY 10003, USA; 2 The Department of Psychology, University of Washington, Seattle, Washington, 98195, USA

## Abstract

**Background:**

Because a large proportion of patients with panic attacks receiving approved pharmacotherapy do not respond or respond poorly to medication, it is important to identify additional therapeutic strategies for the management of panic symptoms. This article describes a randomized, rater-blind study comparing low-dose risperidone to standard-of-care paroxetine for the treatment of panic attacks.

**Methods:**

Fifty six subjects with a history of panic attacks were randomized to receive either risperidone or paroxetine. The subjects were then followed for eight weeks. Outcome measures included the Panic Disorder Severity Scale (PDSS), the Hamilton Anxiety Scale (Ham-A), the Hamilton Depression Rating Scale (Ham-D), the Sheehan Panic Anxiety Scale-Patient (SPAS-P), and the Clinical Global Impression scale (CGI).

**Results:**

All subjects demonstrated a reduction in both the frequency and severity of panic attacks regardless of treatment received. Statistically significant improvements in rating scale scores for both groups were identified for the PDSS, the Ham-A, the Ham-D, and the CGI. There was no difference between treatment groups in the improvement in scores on the measures PDSS, Ham-A, Ham-D, and CGI. Post hoc tests suggest that subjects receiving risperidone may have a quicker clinical response than subjects receiving paroxetine.

**Conclusion:**

We can identify no difference in the efficacy of paroxetine and low-dose risperidone in the treatment of panic attacks. Low-dose risperidone appears to be tolerated equally well as paroxetine. Low-dose risperidone may be an effective treatment for anxiety disorders in which panic attacks are a significant component.

**Trial Registration:**

ClinicalTrials.gov Identifier: NCT100457106

## Background

As described in the DSM-IV, panic attacks are discrete, paroxysmal episodes of intense fear and discomfort accompanied by both somatic and cognitive symptoms, which occur in the absence of actual physical danger [1]. Panic attacks are known to occur in a variety of psychiatric and medical disorders [2], and effective treatment for panic symptoms is important for a large number of patients. Research on the neurobiology of panic has demonstrated a role for both serotonergic and noradrenergic neurotransmitter systems [3-5]. Selective serotonin reuptake-inhibitors (SSRIs) are currently the most widely used and effective pharmacologic agents in the treatment of panic attacks [6,7]. Currently, the U.S. Food and Drug Administration has approved the use of fluoxetine, paroxetine, and sertaline for the treatment of panic disorder [8]. The utility and effectiveness of paroxetine for treating panic disorder and relieving panic attacks has been shown in multiple randomized and controlled trials [9-15]. Additionally, tricyclic antidepressants, monoamine oxidase inhibitors, benzodiazepines, and anti-convulsants have all been used in the treatment of panic disorders with varying and generally reduced degrees of success [16-18]. Despite a multitude of available treatment options, a substantial fraction of patients with panic symptoms do not respond or respond poorly to medication. Between 50 and 80% of patient with panic disorders receiving approved pharmacotherapy continue to have panic attacks and/or avoidance symptoms [12,19,20]. Another study concluded that between 25 – 50% of patients will experience a relapse of symptoms while receiving medication [21]. Thus, it is important to identify additional therapeutic strategies for the management of panic.

Risperidone was approved by the FDA for treatment of psychosis and schizophrenia in 1993. Risperidone has receptor blocking activity at both the D2 receptor family and serotonin receptors, and was therefore considered a theoretical anxiolytic agent. Since then, risperidone has been shown to have anxiolytic effects in patients with schizophrenia and schizoaffective disorder [22,23]. Additionally, risperidone has been shown to be effective in the treatment of anxiety arising in a variety of clinical settings: depression with comorbid anxiety [24,25], treatment resistant anxiety in the elderly [26], generalized anxiety disorder [27], obsessive-compulsive disorder [28-31], and post-traumatic stress disorder [32-35]. Many of these studies reported the anxiolytic effect of risperidone occurred at substantially lower doses than those used in the treatment of psychosis, with a concomitant reduction in the incidence of adverse effects [32,27,25].

Based on these positive findings and the results of our own preliminary study [25], we felt that a trial of risperidone for the treatment of panic attacks was warranted. We describe herein an exploratory, randomized, single-blind trial of risperidone versus paroxetine for the treatment of panic attacks. We hypothesized that risperidone will be as effective as paroxetine in relieving panic symptoms. Because of the low dose of risperidone used in this study, we further hypothesized that risperidone would be better tolerated than paroxetine. To our knowledge, this is the first report of the use of risperidone as monotherapy in the treatment of panic symptoms.

## Methods

This randomized, rater-blinded, medication-controlled study was reviewed and approved by the Beth Israel Medical Center Institutional Review Board, and all subjects provided written, informed consent to participate. The study is publicly registered at ClinicalTrials.gov (Identifier: NCT100457106) .

### Subjects

The study subjects were recruited from the inpatient psychiatric units at Beth Israel Medical Center (BIMC) in New York City, from the Psychiatric Outpatient Service for Adults (POSA) at BIMC, and from ads in local newspapers and on the internet website "craigslist.org" . Inclusion criteria for participation were: 1) males and females, ages 21 – 55; 2) a history of panic attacks; 3) a DSM-IV diagnosis of Panic Disorder, with or without agoraphobia; or Major Depressive Disorder with Panic Attacks, single episode, recurrent, or chronic; [36-39] 4) a Hamilton Anxiety Rating Scale (HAM-A) score of at least 17; and 5) ability to sign informed consent. The initial diagnosis was determined by psychiatric interview conducted by experienced BIMC staff psychiatrists. Because of the frequent comorbidity of panic attacks and Major Depressive Disorder, we chose to include patients diagnosed with Major Depressive Disorder [37,38,40-42]. We required patients to be between the ages of 21 and 55 years old so as to avoid possible confounding effects of including adolescent and geriatric patients. A baseline Ham-A score of at least 17 was required to exclude patients who were asymptomatic and would not therefore demonstrate improvement with any medication. Patients with a current or lifetime history of any Axis I diagnosis other than Panic disorder or Major Depressive Disorder with Panic Attacks were excluded. Participants were also excluded for 1) a history of alcohol or substance abuse within the 6 months preceding enrollment; 2) use of any antipsychotic medications in the two months preceding enrollment; 3) a history of changes in antidepressant or mood stabilizer dosing during the two months preceding enrollment; 4) use of psychoactive medications other that risperidone or paroxetine during the study period; or 5) a history of adverse reaction to either risperidone or paroxetine. All interested subjects who met the inclusion and exclusion criteria were entered into the study after signing an IRB-approved consent form.

### Procedures

Randomization of cases was accomplished using SPSS ver 12.0.1 (SPSS Inc., Chicago Ill.). All subjects were randomly assigned to receive either risperidone or paroxetine on a 1:1 basis. Treatment with risperidone was initiated at 0.25 mg/day. For subjects who did not achieve a remission of panic symptoms, the dose was increased to 0.5 mg once a day on day 3. For subjects who experienced morning sedation, the dose was decreased to 0.125-mg once a day on day 3. Further dosage adjustment for lack or response or sedation was accomplished in 0.25 mg increments as needed. Following conventional treatment guidelines [43], treatment with paroxetine was initiated at 30-mg/day. For the subjects who did not achieve a remission of panic symptoms, the dose was increased as needed. Maximal allowable doses of risperidone and paroxetine were 16 mg/day and 60 mg/day, respectively. Treatment with adjunct psychotropic medications during the study period was not allowed.

The study period was eight weeks in length, during which subjects were assessed at 10 different time points: at the initial interview before receiving any study medication, on study medication day three, on study medication day seven, and once a week thereafter for a total of eight weeks. Subject assessments were conducted by a rater blinded to medication status. The assessment battery included both clinician and patient-rated measures.

• The 17-item Hamilton Depression Rating Scales (HAM-D-17) [44-46]. The Ham-D-17 is a clinician-rated scale, which provides a measurement of symptoms of depression. The Ham-D 17 was administered at all study visits. The total scores were used for analysis.

• The Hamilton Anxiety Rating Scale (HAM-A) [47]. The Ham-A is a 14 item clinician-rated measure, which assesses symptoms of anxiety. The Ham-A was administered at all study visits. The total scores were used for analysis.

• The Panic Disorder Severity Scale (PDSS) [48]. The PDSS is a seven-item, clinician-rated assessment of the symptoms of panic attacks. It was administered at all study visits. PDSS question 1 (panic attack frequency), question 2 (panic attack severity), and PDSS total score were analyzed separately.

• The Sheehan Panic Anxiety Scale-Patient (SPAS-P) [49]. A rating scale completed by patients, comprised of 35 items which provides a measure of anxiety symptoms. It was administered at all study visits. The total scores were used for analysis.

• The Clinical Global Impressions Scale (CGI) [50]. The CGI is a physician administered, single item rating scale that provides an overall assessment of the patients' condition. The CGI was administered at the initial and final study visit.

At the end of study period, participants were given the option to continue their study medications under the care of one of the study physicians or another psychiatrist at POSA. Those wishing to discontinue the study medications at the end of the study were able to do so under the supervision of one of the study physicians. Paroxetine was tapered over a two-week period to avoid SSRI withdrawal. Risperidone was stopped abruptly as no withdrawal symptoms have been reported in the literature.

### Data Analysis

Assignment to a treatment group was carried out using a 1:1 randomization. Group differences in baseline demographic data were assessed using Chi-squared analysis for categorical variables, and t-tests for continuous variables. Data from each outcome measure was checked against the normal distribution using the Kolmogorov-Smirnov test of normality. Data from all outcome measures approximated a normal distribution, with the exception of the CGI, and subscores 1 and 2 from the PDSS. Data normalization for items 1 and 2 of the PDSS was carried out using the following formula: transformed value = log *n *(original value + 1). Because the CGI is rated on a discrete 7 point scale, CGI results were treated as a categorical variable with seven values, and the comparison of CGI results between groups was assessed using Chi-squared analysis. An independent samples t-test was used to compare group means at baseline for each continuous outcome measure. Analysis of the over-time change in clinical measures was assessed using a general linear model for repeated measures as implemented in SPSS ver 12.0.1 (SPSS Inc., Chicago Ill.). All subjects were entered on an intent-to-treat basis, with last observation carried forward (LOCF) for subjects who terminated study participation prematurely [51,52]. Subject attrition was analyzed by treatment group membership using a life table analysis. We then used a Gehan's Generalized Wilcoxon statistic to test the null hypothesis that study attrition is equal for all groups [53].

## Results

### Subject Characteristics

Fifty-six subjects were enrolled in the study: 40 female, and 16 male (71 and 29%, respectively). Thirty three subjects were randomized to receive risperidone, and 23 received paroxetine (57% and 43%, respectively). In order to test whether the observed randomization distribution deviated significantly from the expected distribution (i.e. 28 subjects in each treatment group), we computed the binomial sampling calculation. With a sample size of 56, and a random assortment probability of 0.5, the mean and SD of the binomial sampling distribution is 28 and 3.74, respectively. The exact binomial calculation (one-tailed) of the probability of an assortment of 33 or greater in this sample of 56 is 11.44%. This suggests to us that the observed subject randomization occurs within a statistically reasonable frequency.

Subject characteristics are presented in Table 1. The average age for all subjects was 40.36 ± 12.37 years. Forty three subjects were diagnosed with Panic Disorder, and thirteen were diagnosed with major depressive disorder with panic attacks (76.8% and 23.2%, respectively). The treatment groups did not differ significantly in age, gender make-up, or psychiatric diagnosis. The average dose for the risperidone group was 0.53 mg. (range 0.125 – 1.0 mg.); for the paroxetine group, all subjects received a dose of 30 mg. except one subject who was given a final dose of 40 mg.

**Table 1:** Demographics

	**Risperidone**	**Paroxetine**	**Statistics**
N	33	23	

Gender			χ^2 ^= 0.74; n.s.
Males (%)	8 (24)	8 (35)	
Females (%)	25 (76)	15 (65)	

Diagnosis			χ^2 ^= 0.74; n.s.
Panic Disorder (%)	24 (73)	19 (83)	
MDD (%)	9 (27)	4 (17)	

Age: mean (± SD)^1^	38.82 (9.74)	42.57 (14.34)	*t *= 1.16; n.s.

Terminated (%)^2^	13 (39)^4^	14 (61)^4^	χ^2 ^= 2.50; n.s.
LTF/Non-compliance (%)	9 (69)^5^	5 (36)^5^	χ^2 ^= 0.87^3^; n.s.
Adverse Effects (%)	2 (15)^5^	1 (11)^5^	
Lack of Response (%)	2 (15)^5^	1 (11)^5^	

Demographic data for study subjects. Percentages represent within-group proportion

^1 ^Age in years

^2 ^Number of subjects failing to complete the study.

^3 ^Testing the difference between groups for the reason for attrition

^4 ^Percentage of all subjects within treatment group

^5 ^Percentage of terminated subjects within treatment group

n.s. – not statistically significant

LTF – lost to follow-up

### Retention

**Figure 1:**
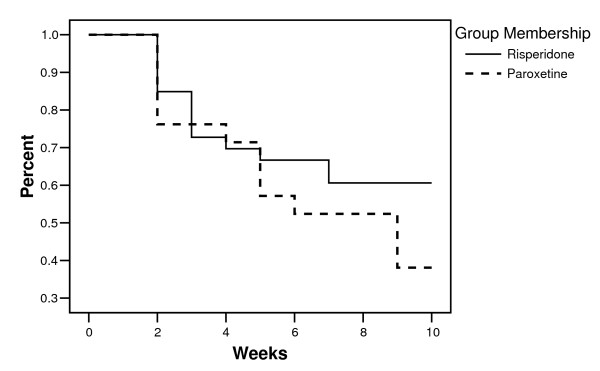
**Survival by Group**. Subject study attrition by treatment group.

Twenty nine subjects completed all 10 visits in the study period, and 27 dropped out prematurely (51.8% and 48.2%, respectively). In the risperidone group, 20 subjects (60.6%) completed all 10 study visits, and 13 subjects (39.4%) dropped out prematurely. In the paroxetine group, nine subjects (39.1%) completed all 10 study visits, and 14 subjects (60.9%) dropped out prematurely. By chi-square analysis, there was no statistical difference between the proportions of subjects in each group that did not complete the study. Subject retention over the course of the study for each group was examined using the Wilcoxon (Gehan) statistic. For group membership the statistic value is 1.28, and is not statistically significant (Figure 1). This indicates that the survival curves over the course of the study did not differ statistically between the two treatment groups. Reasons for attrition from the study were: 1) lost to follow-up/non-compliance (n = 14; 51.9%); 2) intolerable side effects (n = 3; 11.1%); 3) lack of therapeutic response (n = 3; 11.1%). The reason for attrition was not collected for seven subjects who did not complete the study. Reasons for attrition from the study were examined for each treatment group: the Pearson's chi-square value is 0.87 and is not significant.

### Outcome Measures – Baseline

Initial scores on the CGI, Ham-A, SPAS-p, PDSS total, PDSS item 1, and PDSS item 2 did not differ across groups (See Table 2). Baseline scores on the Ham-D were significantly greater (more severe symptoms) for the paroxetine group compared to the risperidone group. Initial scores on the SPAS-p showed a greater mean (more severe symptoms) for the risperidone group, compared to the paroxetine group, and this difference was close to, but did not attain, statistical significance. See Table 2.

**Table 2:** Outcome measures at baseline and at the final assessment.

	**Baseline (V1)**	**Final (V10)**
**CGI**		
Risperidone	4.40 (± 0.60)	2.84 (± 1.02)
Paroxetine	3.81 (± 1.33)	2.67 (± 0.71)
Statistics^3^	χ^2 ^= 7.10, df = 5, n.s	

**PDSS**		
Risperidone	13.5 (± 4.42)	7.87 (± 5.87)
Paroxetine	12.38 (± 6.77)	8.35 (± 6.15)
Statistics^3^	*t *= 0.715, df = 49, n.s.	

**PDSS question 1^1^**		
Risperidone	1.16 (± 0.058)	0.70 (± 0.088)
Paroxetine	0.94 (± 0.072)	0.60 (± 0.11)
Statistics^3^	*t *= 1.77, df = 29.47, *p *= n.s.	

**PDSS question 2^2^**		
Risperidone	2.41 (± 0.875)	1.28 (± 1.17)
Paroxetine	2.39 (± 1.27)	1.52 (± 1.12)
Statistics^3^	*t *= 0.049, df = 36.55, n.s.	

**Ham-A**		
Risperidone	25.09 (± 5.27)	13.75 (± 9.67)
Paroxetine	27.76 (± 7.75)	16.22 (± 9.51)
Statistics^3^	*t *= 1.49, df = 51, n.s.	

**SPAS-p**		
Risperidone	72.53 (± 27.51)	74.69 (± 24.6)
Paroxetine	87.52 (± 25.46)	84.61 (± 30.12)
Statistics^3^	*t *= 2.00, df = 51, *p *= 0.051	

**Ham-D**		
Risperidone	26.19 (± 8.37)	17.52 (± 10.91)
Paroxetine	31.0 (± 8.74)	21.68 (± 11.79)
Statistics^3^	*t *= 2.01, df = 51, *p *= 0.049	

Initial and final assessments for all outcome measures.

1 – panic attack frequency

2 – panic attack severity

3 – testing the difference in baseline scores between treatment groups

### Outcome Measures – CGI

**Figure 2:**
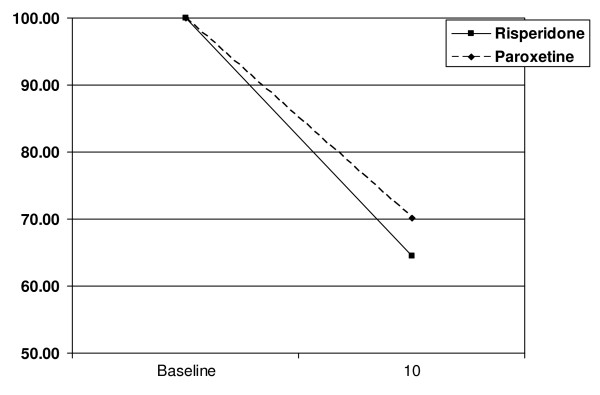
**CGI Scores Over Time**. CGI scores at baseline and at termination by treatment group, as a percent of baseline score. Scores for subjects terminating study treatment prematurely were carried forward to V10.

All subjects demonstrated a decrease in CGI scores over the course of the study, regardless of treatment. There was no difference in the over-time change in CGI scores between groups. For all subjects together, there was a significant decrease in CGI scores between the first and the last assessment (Wilcoxon signed rank test Z = 4.15, p < 0.001). Group differences in the over-time change in CGI scores were calculated by subtracting the last CGI score from the initial CGI score, and comparing the results across treatment groups. The difference between groups in the over-time change in CGI scores was not significant (χ^2 ^= 7.45; df = 4; *p *= 0.114). The change in CGI scores are graphically represented in Figure 2.

### Outcome Measures – PDSS, Ham-A, and Ham-D

**Figure 3:**
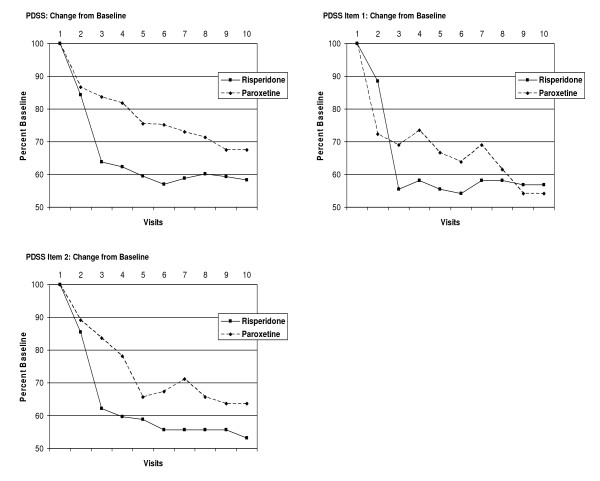
**PDS Scores Over Time for Risperidone and Paroxetine Treatment Groups**. Scores over time for Total PDSS, PDSS Item 1, and PDSS Item 2 for two treatment groups, as a percent of baseline score. Scores for subjects terminating study treatment prematurely were carried forward to V10.

**Figure 4:**
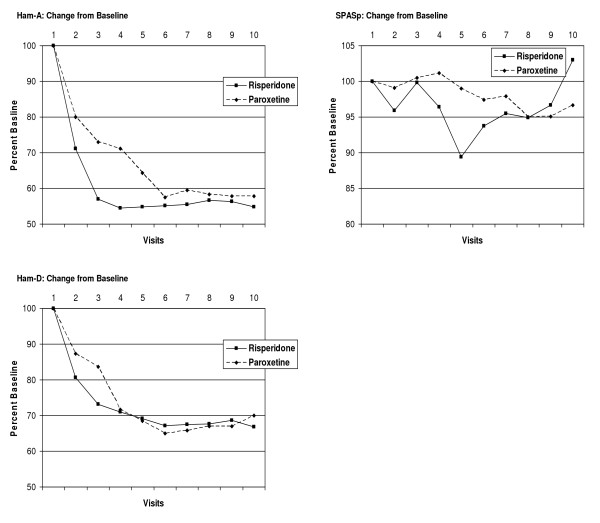
**Ham-A, Ham-D, and SPASp Scores Over Time for risperidone and Paroxetine Treatment Groups**. Scores over time for Total Ham-A, Total Ham-D, and Total SPASp for two treatment groups. Scores for subjects terminating study treatment prematurely were carried forward to V10.

For the measures PDSS total score, PDSS item 1, PDSS item 2, Ham-A, and Ham-D, all subjects, regardless of treatment group, demonstrated a significant decrease in outcome scores during the course of the study. By the end of the study period, there was no statistical difference between the treatment groups in the total scores. The omnibus test demonstrated a significant change in total scores over time but no significant effect of group membership on the change over time. The tests of the within-subject contrasts comparing measurements made at each visit with scores at baseline were all significant at the level of p < 0.005 or lower, with the exception of the contrast of visit 1 with visit 2 for PDSS total score, which was significant at *p *= 0.037. This data is presented graphically in Table 3, and Figures 3 and 4.

**Table 3:** Repeated Measures Testing

	**Effect of Time**	**Effect of Treatment on Time**
**PDSS**		
All Subjects	F (9,40) = 3.71; *p *= 0.002	F (9, 40) = 1.11; *p *= 0.10
Low Ham-D group^1^	F (9,14) = 2.69; *p *= 0.047	F (9,14) = 2.01; *p *= 0.12
High Ham-D group^2^	F (9,16) = 1.98; *p *= 0.11	F (9,16) = 0.22; *p *= 0.99

**PDSS q1**		
All Subjects	F (8,44) = 4.42; *p *< 0.001	F (8,44) = 1.41; *p *= 0.22
Low Ham-D group^1^	F (8,17) = 2.34; *p *< 0.067	F (8,17) = 1.85; *p *= 0.14
High Ham-D group^2^	F (8,18) = 1.75; *p *= 0.15	F (8,18) = 0.45; *p *= 0.87

**PDSS q2**		
All Subjects	F (8,44) = 5.16; *p *< 0.001	F (8,44) = 1.25; *p *= 0.29
Low Ham-D group^1^	F (8,18) = 5.19; *p *< 0.002	F (8,18) = 0.85; *p *= 0.57
High Ham-D group^2^	F (9,18) = 2.3; *p *= 0.064	F (9,18) = 1.36; *p *= 0.28

**Ham-A**		
All Subjects	F (8,43) = 8.81; *p *< 0.001	F (8,43) = 1.90; *p *= 0.077
Low Ham-D group^1^	F (9,15) = 4.24; *p *= 0.007	F (9,15) = 1.96; *p *= 0.12
High Ham-D group^2^	F (9,18) = 5.05; *p *= 0.002	F (9,18) = 0.75; *p *= 0.66

**SPAS-p**		
All Subjects	F (9,41) = 0.80; *p *= 0.62	F (9,41) = 0.57; *p *= 0.82

**Ham-D**		
All Subjects	F (9,43) = 4.30; *p *< 0.001	F (9,43) = 1.17; *p *= 0.33

Outcome measures tested by a General Linear Model Repeated Measures analysis. Testing was initially done for all subjects together, and then for subjects grouped into high or low Ham-D groups based on initial Ham-D scores.

1 – Subset of patients with Ham-D scores < 27

2 – Subset of patients with Ham-D scores ≥ 27

### Outcome Measures – SPAS-p

There was little change in SPAS-p scores over time, regardless of treatment group. Average SPAS-p scores were lower in the risperidone group compared to the paroxetine group throughout the course of the study, but this difference was non-significant. The omnibus test demonstrated no significant change in SPAS-p scores over time, and no significant effect of group membership on the change over time. The change in SPAS-p scores are graphically represented in Table 3 and Figure 4.

### Post Hoc Studies

Based on our observation of treatment group differences in outcome scores during the initial weeks of the trial, we performed independent sample t-tests to compare outcome measures at visits 3 and 4 across treatment groups. For the Ham-A, the risperidone group had a significant reduction in the mean visit 3 score (14.31 ± 9.83) compared to the paroxetine group (20.3 ± 9.26), where t = 2.28, and *p *= 0.026. Additionally, the risperidone group demonstrated a significant reduction in the mean Ham-A visit 4 score (13.69 ± 9.63) compared to the paroxetine group (19.78 ± 8.33) (t = 2.25, p = 0.018). For the Ham-D measure, the risperidone group again had a lower mean score at visit 3 compared to the paroxetine group (19.15 ± 11.16 and 25.95 ± 11.72, respectively). This difference was significant (t = 2.17, p = 0.034). Although the risperidone group had a lower mean visit 4 Ham-D score than the paroxetine group (18.61 ± 11.28 vs. 22.23 ± 11.79), this difference was not statistically significant (t = 1.15, p = 0.26). For the PDSS total score, the difference across treatment groups in mean visit 3 and mean visit 4 scores were not significant (visit 3: t = 1.10, p = 0.28; visit 4: t = 1.02, p = 0.31). Total PDSS scores for the risperidone and paroxetine groups, respectively, were 8.61 ± 5.58 and 10.35 ± 5.59 on visit 3 and 8.41 ± 6.0 and 10.13 ± 6.32 on visit 4. In summary, compared to subjects receiving paroxetine, subjects receiving risperidone had significantly reduced symptoms as measured by the Ham-A at visits 3 and 4, and by the Ham-D at visit 3.

Our results show that baseline Ham-D scores were significantly higher in the paroxetine group than in the risperidone group, potentially confounding any treatment effect. Additionally, we found significant positive correlations of baseline Ham-D scores with both baseline and outcome measures of anxiety symptoms: the correlation of baseline Ham-D with baseline Ham-A scores was 0.541 (p < 0.001), with V5 Ham-A scores was 0.439 (p = 0.001), with V10 Ham-A scores was 0.381 (p = 0.005), with baseline total PDSS was 0.259 (p = 0.067), with V5 total PDSS scores was 0.379 (p = 0.006), and with V10 total PDSS scores is 0.246 (p = 0.076). With this positive correlation of baseline Ham-D and key outcome measures, covarying baseline Ham-D scores in the repeated measure analysis has the mathematical effect similar to covarying the outcome measure with itself, rendering such an analysis meaningless. Indeed, when the subjects' baseline Ham-D scores were entered as covariates in the statistical analysis, treatment effects for all subjects were reduced in significance (see Table 3).

As an alternative, we elected to stratify all subjects by baseline Ham-D score and redo the repeated measures analysis for each sub-group. All subjects were divided into high and low Ham-D groups as determined by their baseline Ham-D score. Using the median baseline Ham-D score of 26.0 as the cut-off, there were 25 subjects classified as low Ham-D, and 30 subjects classified as high Ham-D. In subjects receiving risperidone, low and high Ham-D subjects were 51.5% and 48.52%, respectively; for subjects receiving paroxetine, low and high Ham-D subjects were 36.4% and 63.6%, respectively. The proportion of low and high Ham-D subjects did not differ significantly across treatment groups (χ^2 ^= 1.22; df = 1; *p *= 0.27). The repeated measures test for PDSS and Ham-A scores were then retested on each sub-group (see Table 3). These results are consistent with the findings of our primary analysis done for all subjects together.

## Discussion

This preliminary study compares the effectiveness of risperidone and paroxetine for the treatment of panic attacks. We found that both risperidone and paroxetine were effective in reducing the occurrence and severity of panic attacks. Additionally, there was no difference in the efficacy of risperidone and paroxetine in ameliorating the symptoms of anxiety associated with panic disorders, as evidenced by the improvements over time in the scores of the Ham-A, Ham-D, PDSS, and CGI. As measured by retention in the study, treatment with risperidone appeared to be tolerated as well as paroxetine; there was no difference in retention between the two treatment groups. These results suggest that low-dose risperidone is an effective treatment for panic attacks and anxiety in individuals with panic attacks.

We have presented evidence that suggests that treatment with risperidone results in a faster symptomatic relief than does treatment with paroxetine. Group means of the total PDSS, panic attack frequency, panic attack severity, and Ham-A all showed a faster decline in the risperidone group than in the paroxetine group. However, using a general linear model repeated measures test, these differences did not achieve statistical significance. We suspect this difference was not demonstrated statistically because our study was not sufficiently powered. Using less restrictive *t*-tests to examine group differences at specific points of time, we demonstrated a statistically significant decrease in scores of the Ham-A at visits three and four, and scores of the Ham-D at visit three, in the risperidone group compared to the paroxetine group. However, no group differences in PDSS scores were identified at either visit three or four. Confirmation of a faster treatment response using either medication may require a study using a larger sample group.

The observed reduction of anxiety symptoms in this sample is potentially confounded by the presence of the diagnosis of major depressive disorder in 23% of our subjects. Additionally, as demonstrated by the post hoc analyses, there was a positive correlation of Ham-D scores with Ham-A scores, and to a lesser extent PDSS scores. We therefore can not know if the anxiolytic effect of risperidone is secondary to a concomitant reduction in symptoms of depression. We attempted to examine the effect of baseline depressive symptomatology on anxiolytic efficacy of the medications under study by stratifying our subjects into high and low depression groups and re-testing the change in anxiety scores. Both the subjects identified as having high depressive symptoms and low depressive symptoms at baseline demonstrated a similar anti-anxiety response to the two study medications. We conclude that in our sample of subjects the anxiolytic response to the study medications was no different in subjects with more depressive symptoms than in subjects with fewer depressive symptoms.

Previous studies of risperidone in anxiety disorders have focused on generalized anxiety disorder, obsessive-compulsive disorder, and post-traumatic stress disorder [54]. To the best of our knowledge, this is the first study to examine the effectiveness of risperidone in treating panic. Our results in patients with panic attacks are consistent with previous studies of the effectiveness of risperidone in other anxiety disorders. Brawman-Mintzer [27] reported that adjunctive risperidone was more effective than placebo in subjects suffering with generalized anxiety disorder. Adjunctive risperidone was also reported to be effective in reducing symptoms of post-traumatic stress disorder as well as scores of the Ham-A and the positive subscale of the Positive and Negative Syndrome Scales (PANSS) in patients diagnosed with post-traumatic stress disorder [32]. Risperidone augmentation has been shown to be effective in reducing anxiety symptoms in a group of patients with a variety of anxiety disorders refractory to adequate treatment with antidepressants and/or benzodiazepines [55]. In a study of risperidone monotherapy, risperidone was reported to be more effective than placebo in reducing anxiety in women diagnosed with PTSD [56]. Our results provide further evidence of the utility of risperidone as an anxiolytic agent. Moreover, our study suggests that this anxiolytic activity is present when risperidone is used as monotherapy.

It is interesting to note that the one measure that did not show improvement over the eight week study period was the SPAS-P. This is a patient-rated measure, the only one used in this study. All clinician-rated measures demonstrated improvement over the treatment period. Taken together, the results from all the outcome measures suggest that clinician/raters perceive improvement when the patients themselves do not. It is not uncommon in clinical practice to see evidence of a response to treatment before the patients' subjective sense of well-being improves. It may be that with a longer duration of the study period, we would see improvement in the SPAS-P reflecting the patients' perception of symptomatic improvement.

Risperidone is an antagonist at both the dopamine D2 receptor family (D2L, D2S, D3, and D4), and the 5-HT_2A _receptors [57-59]. Interaction of risperidone with the dopamine D1 receptor only occurs at very high concentrations. It is the serotonin 5-HT_2A _and dopamine D2 receptor occupancy that seem to provide the anti-psychotic effects of risperidone. Studies in lab animals have demonstrated that induced anxiety increases the release of dopamine in the prefrontal cortex, amygdala, and other brain areas [60-63]. Both typical and atypical antipsychotic medications have been shown to block the acquisition of conditioned fear behavior [64-66]. These data suggest that risperidone may alleviate anxiety symptoms through the direct modulation of the dopamine system [54]. Furthermore, it has been suggested that the anti-anxiety effect of selective serotonin reuptake inhibitors (SSRIs) is due in part to blockade of excitatory 5-HT_2A _receptors located on inhibitory γ-aminobutyric acid (GABA) interneurons, in turn suppressing the firing of noradrenergic neurons in the locus ceruleus [67,68]. Indeed, the affinity of risperidone for the 5-HT_2A _receptor is greater than its affinity for D2 receptors [54,69]. Thus, our data suggest that the dual action of risperidone in suppressing both dopaminergic and serotonergic activity may be beneficial for an anxiolytic effect.

One reason we were interested in studying risperidone as a treatment for panic attacks is our belief that symptomatic relief may occur using very low doses, with a concomitant reduction in adverse effects. Our results show that risperidone has effective anxiolytic properties at doses far below those used in treating psychosis. The average risperidone dose in our subjects was 0.53 mg/day, and no patient required treatment with greater than 1.0 mg/day; a dose less than one-half that typically used for treating psychosis. Few subjects complained of adverse effects to either of the medications under study. Two subjects in the risperidone group and one subject in the paroxetine group complained of adverse effects. This difference was not statistically significant, and suggests that the acceptance of risperidone in our sample is comparable to that of paroxetine. Our study of subject attrition during the course of the study showed no difference between the two treatment groups in attrition, and gives further evidence that subjects' tolerability to risperidone did not differ from that of paroxetine.

The results of this study should be considered in conjunction with its limitations. Our study was limited by the short 8 week follow-up period that does not provide for data on the long-term efficacy and safety of the use of risperidone in patients with panic attacks. Additionally, our report is limited by the loss of data because of subject attrition, which in this study amounted to 48% of the total sample. As such, our study perhaps better mirrors the experience of clinicians in an office practice where patients frequently fail to return for appointments [70,71]. Baseline Ham-D scores differed between treatment groups; though we have tried with our post-hoc testing to elucidate the possible significance of this difference, the overall significance is unknown. The initiation of medications was titrated for Risperidone, but no titration was used for the Paroxetine group, possibly confounding treatment and survival data. We did not use a standardized diagnostic instrument verify the subjects' diagnoses. Because we studied patients with both MDD and PD, we cannot make firm conclusions about the effectiveness of Risperidone as a treatment for PD. Additionally, it is possible that subjects with MDD and PD will have different responses to the study medications, and our results are confounded by these as yet unreported differences. Lastly, we did not record tobacco use among our sample, and given the reported link between cigarette smoking and panic attacks [72], our results may be confounded by smoking in unknown ways.

## Conclusion

The strengths of our study are the prospective, randomized design, the use of risperidone as monotherapy, the use of an effective comparator medication, and the multiple measures of anxiety symptoms employed. In it we have shown that low-dose risperidone provides effective relief of panic attacks and concomitant symptoms of anxiety. Risperidone appears to be well-tolerated. In addition, it is possible that low-dose risperidone results in faster symptomatic relief than paroxetine. Further studies involving larger groups are necessary to confirm these results.

## Competing interests

The authors declare that they have no competing interests.

## Authors' contributions

IG designed the study and wrote the protocol. JP and LC participated in data analysis, interpretation, and manuscript writing. SY participated in data collection. All authors read and approved the final manuscript.

## Pre-publication history

The pre-publication history for this paper can be accessed here:


